# End of life care in the setting of extreme prematurity – practical challenges and ethical controversies

**DOI:** 10.1016/j.siny.2023.101442

**Published:** 2023-08

**Authors:** Dominic JC. Wilkinson, Sophie Bertaud

**Affiliations:** a Oxford Uehiro Centre for Practical Ethics, Faculty of Philosophy, University of Oxford, UK; b John Radcliffe Hospital, Oxford, UK; c Murdoch Children's Research Institute, Melbourne, Australia; d Centre for Biomedical Ethics, National University of Singapore Yong Loo Lin School of Medicine, Singapore

**Keywords:** Premature infant, Decision-making, End of life, Palliative care, Intensive care, Neonatal

## Abstract

While the underlying principles are the same, there are differences in practice in end of life decisions and care for extremely preterm infants compared with other newborns and older children. In this paper, we review end of life care for extremely preterm infants in the delivery room and in the neonatal intensive care unit. We identify potential justifications for differences in the end of life care in this population as well as practical and ethical challenges.

## Highlights

• Consider all relevant prognostic factors (not just gestational age) in decision-making about resuscitation at birth for extremely preterm infants (EPI)
• Where palliative care is planned in the delivery room, the presence of experienced neonatal staff may support parallel planning and symptom management
• Non-pharmacological measures should always be provided for EPI receiving palliative care (in the delivery room or NICU). Buccal or nasal opioids may be of value where infants do not have vascular access.
• Where intensive care has been initiated, and infants fail to respond or develop complications, consider the option of withdrawal of treatment in situations where withholding of treatment (if not started) would be ethical.

## 1 Research agenda

• There is a need for better characterisation of symptoms and experience for EPI dying in the delivery room and in the NICU
• There is a need for prospective studies evaluating the perceptions of parents about End of Life Care (EOLC) for EPI
• Prospective studies are needed to evaluate different approaches to symptom management in EPI receiving EOLC

The fundamental principles underlying End of Life Care (EOLC) for extremely premature infants (EPI) are the same as those underpinning the care of older infants, children and adults. That might imply that there is little need to discuss EOLC for this population separately. However, in practice there are often differences in how decisions are reached and how EPI die. The aim of this paper will be to identify some of these differences, to examine whether they are ethically justified and to highlight important ongoing sources of difficulty and controversy. We will discuss elements of both end of life decision-making and care for these tiniest patients.

Extreme prematurity is traditionally defined as those infants born prior to 28 weeks gestation [1]. Such infants are a minority of preterm births, and constitute only ∼0.5% of overall births [1]. Yet in high income settings, because of their long average length of stay, EPI often constitute a large proportion of infants in Neonatal Intensive Care Units (NICU). They also contribute disproportionately to deaths (approximately 40% of deaths in perinatal units) [2]. Amongst EPI, there are also differences. More than 80% of infants born at 26 or 27 weeks survive, but there is an extremely high mortality rate (70–80%) at the lowest gestation that babies are admitted to the NICU, i.e. at 22 weeks gestation [1,3]. There are also liveborn infants born earlier than this (i.e. at 20 or 21 weeks gestation) for whom the mortality rate is virtually 100%.

EOLC for extremely premature infants might be divided into that occurring in the delivery room, and in the NICU. We will discuss these separately.

## 2 End of life care in the delivery room for extremely premature infants

### 2.1 Decision-making

Antenatal counselling and decision-making in the setting of anticipated extremely preterm birth is an extremely familiar, even routine part of practice in the NICU. It is standard for neonatal health care professionals to meet with parents prior to such a delivery and to make plans about the care of the baby when they are born [4]. Many parts of the world have developed guidance for identifying when active survival-focused care should almost always be provided, when it would not be appropriate to do so (because the outlook is too poor), and when it may be appropriate to provide either active survival focused care or palliative comfort focused care depending on the views of parents [3,5]. This gives rise to the concept of the neonatal “grey zone”: cases where parental decisions prior to birth will determine whether end of life care is provided for an extremely premature infant at delivery [6]. If a delivery is perceived to lie within the grey zone, antenatal counselling may be particularly important because of the potential for making an end of life decision.

One important practical question for any anticipated extremely preterm delivery is whether this lies within the grey zone or not.

Case 1 :A mother is in threatened preterm labour at 24 + 1 weeks gestation, expecting a female fetus with an estimated fetal weight of 620 g. The mother has received two doses of antenatal steroids. The obstetric team have arranged for a paediatrician to provide counselling. Is this delivery in the grey zone? Should the paediatrician discuss and offer the option of providing comfort care at delivery? If this were requested by the mother, should that choice be respected?

Guidelines relating to these decisions have often used gestational age to identify the boundaries of the grey zone [5,7]. However, such an approach has been criticised as being arbitrary, simplistic and ethically flawed [[7], [8], [9], [10], [11]]. In response to such criticisms, some recent guidelines have shifted to including other factors relevant to an extremely premature infant's prognosis (for example, presence of severe growth restriction, administration of antenatal steroids, place of delivery, singleton vs multiple, fetal sex) [3,12]. There is considerable existing literature relating to antenatal counselling and guidelines relating to decision-making at birth (though trial evidence is almost non-existent) [4,[13], [14], [15]]. However, there remain challenges. For example, should there be a lower gestation limit for initiating active survival focused care in EPI? If prognosis is the basis for decisions about EPI, how should it be determined, and what prognosis would justify providing or withholding treatment? Should the thresholds for treatment be different to those that apply to term or older infants? We return to this last question below.

### 2.2 Care

Case 2 A mother is in preterm labour at 23 + 1 weeks gestation. Following antenatal counselling with the neonatal team she has decided that she wishes her infant to receive palliative care at delivery. Should the neonatal team attend the delivery?, A few hours later, a female infant is born weighing 575 g. She is wrapped and given to the mother to hold. The infant has a heart rate of more than 100, and is breathing regularly. Should the decision to provide palliative care be revisited or revised?, Should the infant receive analgesic or sedative medication?

#### 2.2.1 Attendance

For the most premature infants, those born at 20–21 weeks gestation, it is common that neonatal teams do not attend delivery; this is often endorsed by professional guidance [3]. The same approach might be taken to peri-viable deliveries (e.g. case 2) where there has been a conscious decision to provide palliative care. The potential justification is that neonatal expertise is not required, since active survival-focused care will not be provided. A different (anecdotal) concern is that attendance of neonatal teams might lead to inappropriate initiation of treatment or to moral distress because junior staff are unfamiliar with withholding survival-focused care in the delivery room. Where neonatal teams do not attend, the expectation is that midwifery and obstetric teams will support palliative care of the newborn.

However, that might lead to two challenges.

#### 2.2.2 Parallel planning

One important principle in palliative care is to develop plans for care that acknowledge uncertainty of outcome and are able to flexibly adjust to changing circumstances [16]. In some situations, plans to provide comfort-focused care at delivery for an EPI may be revised after birth because either the infant's condition is significantly better than expected, and/or because the parents change their mind [3]. The former may occur because of natural variability in condition of EPI at delivery, or because there has been a mistake in calculation of gestation. The latter might occur because of parents' response to seeing their newborn infant, or because the infant's condition is different from what they had been expecting. Even parents who have made a clear decision to withhold life-prolonging treatment may experience doubt when the infant is born.

It is important to consider this possibility and it may be helpful to discuss pre-emptively. However, even if forewarned, this may not be easy to address. It may be difficult for midwives to know whether baby's condition is consistent with expectations (e.g. case 2). That is one reason in support of experienced neonatal team members being present. Such professionals can empathically affirm and support the parents' choice of palliative care as well as be present for those (rare) instances where a decision should be revised. However, it is not necessarily easy for neonatal professionals either. Assessing either gestational age or infant condition at delivery is subjective and potentially inaccurate [17]. In situations of genuine uncertainty, there is the possibility of initiating therapy which may subsequently be withdrawn; however, that runs the risk of subjecting the infant to potentially burdensome and non-beneficial therapies.

#### 2.2.3 Symptom management

A separate reason for neonatal teams to be present is to support symptom management as part of EOLC in the delivery room.

There is relatively little data to guide this. It is unclear how often infants have significant symptoms, nor which interventions are effective in relieving them, nor how best to deliver them. Infants being provided with planned palliative care in the delivery room will usually not have vascular access, and attempts to secure such access might separate the infant from parents during their short time together.

A study from Berlin included 86 infants born at 22–23 weeks and receiving palliative care [18]. The infants were perceived to have few or no signs of distress and survived for a median of 60 min. Only two infants received any medication for symptoms. Similarly, studies from US, Netherlands, Switzerland and France found that very few infants who died in the delivery room received comfort medication [[19], [20], [21]]. A somewhat different picture was described as part of a large cohort study from France (the Epipage study). This described 73 EPI ( ≥ 22 weeks gestation) who died in the delivery room. Gasping was described in two thirds, and comfort medicines (mostly opioids or midazolam) were provided in half. Medication was often given via an umbilical catheter, but sometimes rectally [22]. To our knowledge, there is no literature describing symptoms or symptom management in infants born prior to 22 weeks gestation. In a Californian population-based study, approximately one third of infants born at 20–21 weeks gestation were liveborn [23]. An earlier study found that at this gestation liveborn infants survived for a median of 60 min [24].

What should our approach to care be? One uncontroversial suggestion is that non-pharmacological measures should be used first to improve the comfort of EPI receiving EOLC in the delivery room [25]. This would apply to both pre-viable and peri-viable infants. Such measures include provision of wrapping, warmth, and continuous physical touch from parents as well as avoidance of stimuli such as loud noise, bright lights or cold air. In conjunction with this, interventions that might cause discomfort (e.g. physical examination, airway procedures, vascular access) should be minimised or avoided. Administration of oxygen is described in some cases, but it is unclear what role it might play in increasing comfort.

Is more than this necessary? Garten and colleagues have argued that additional measures are not usually required [25]. They note that such infants do not (usually) have disease or iatrogenic processes that would cause physical pain, that the mechanism of dying is usually primary apnoea rather than dyspnoea, and that they may have physiological factors that diminish awareness or perception of discomfort (e.g. hypoxia, hypercapnoea and high vasopressin levels) [18,25]. One additional factor for the most premature infants receiving EOLC in the delivery room may be immaturity in the neural pathways for perception of pain or discomfort, though the significance of this remains controversial [26].

One symptom that has been described in EPI dying in the delivery room is gasping [22]. Deep, infrequent, so-called “agonal” breaths are observed in the terminal phase in patients of all ages [27]. They are thought to be a reflex response to profound hypoxia initiated by the brain-stem. In older populations there is controversy about whether dying patients are conscious of such gasping, and many have concluded that (because of concomitant severe hypoxia) this is unlikely [27]. Nevertheless, it is impossible to rule out some degree of awareness; moreover, such gasping can be very distressing for family members to witness. Some have argued that it may be preferable to err on the side of assuming that the patient is possibly suffering (and treat unnecessarily). For that reason, some have argued in favour of pharmacologically treating gasping in dying patients where family members desire this [27]. It appears that in the Epipage cohort, most medications given were in response to gasping.

If medication is given, whether for gasping or other symptoms, one suggestion has been that, as in other age groups, a reasonable first line would be opioids [25]. Given the lack of vascular access (and a desire to avoid interrupting parental contact with the infant), suggestions have included the use of intranasal fentanyl, buccal diamorphine or oral morphine [28,29].

## 3 End of life care in the neonatal intensive care unit for extremely premature infants

### 3.1 End of life decision-making

In high income settings like the UK and US, two thirds to three quarters of extremely premature infants admitted to neonatal intensive care survive to be discharged [30]. Of those EPI who die, approximately one quarter die in the first 24 h, half die in the first month (after 24 h), while a quarter die late, after more than one month of age [30].

There are significant regional differences in end of life decision-making prior to death [31]. In neonatal units in North America, Europe and Australia, most deaths in neonatal intensive care are preceded by end of life decisions and limitation of life-sustaining treatment [31]. A large prospective study in the US identified that two-thirds of deaths of EPI followed withholding or withdrawal of life-sustaining treatment [32]. However, this is much less common in neonatal units in other regions including South America, East Asia and the Middle East [31].

Decisions to limit treatment for extremely premature infants, as for older infants (and children) may be made on the basis of judgement that the infant is actively dying despite maximal therapy, a prediction that the infant is highly likely to die if treatment were continued, or based on concern for the child's quality of life if they survive [33]. Combinations are possible. The most common causes of death are severe respiratory illness, intraventricular haemorrhage, and sepsis (including necrotising enterocolitis) [34].

There are a range of challenging ethical questions about end-of-life decisions in EPI. Here, we will focus on two of them: first, how should decisions about EOLC in the NICU compare with decisions in the delivery room; and second, how should decisions about EOLC for EPI compare with decisions for term infants or older children.

Case 3 An extremely premature infant is born at 23 + 5 weeks gestation following precipitate delivery and no time for antenatal steroids or counselling. He is managed in intensive care but develops a large pulmonary haemorrhage on day 2 of life requiring significant escalation of cardiac and respiratory support overnight. The following morning he is stable on high levels of respiratory support, but a cranial ultrasound shows a unilateral grade III intraventricular haemorrhage. Should parents be offered the option of withdrawal of intensive care?

#### 3.1.1 Withdrawing versus withholding decisions

Although the grey zone is reasonably well characterised for decisions in the delivery room for EPI, there can be much greater uncertainty and variation in subsequent end of life decisions. Large studies have reported significant variability between neonatal units in the rates of withholding and withdrawing of treatment [32]. One potential reason for this is that it can be difficult for clinicians to decide (and agree) whether the prognosis for an individual infant is poor enough to provide end of life care.

One potential challenge is prognostic uncertainty and clinical heterogeneity. Whereas prognosis prior to delivery for an EPI can be estimated based on a set of well characterised factors [3], subsequent prognosis is more complex [35]. For example, it is difficult to find data that describe the outcome of infants like the one in case 3. Overall, EPI with unilateral grade III intraventricular haemorrhage have a range of outcomes (not necessarily severe) [36]. However, cohort data include many infants who will have been born at more than 23 weeks, and with other more positive prognostic factors.

A different potential barrier to end of life decision-making for EPI is a feeling that once treatment has been initiated it is more difficult or ethically fraught to stop [37]. This is related to a perceived difference between withholding and withdrawing life-prolonging treatment [38]. Many professional guidelines indicate that such decisions are ethically equivalent, since the consequences for patients, the intentions of doctors, and the ethical responsibility of professionals for decisions are all identical [38]. However, there is evidence that many health professionals do not find these decisions to be equivalent; they often judge withdrawal of treatment to be more serious [39]. Neonatologists appear to be more comfortable with withholding treatment in the delivery room than stopping treatment later in intensive care [37]. How should we respond to this problem? That depends in part on what we diagnose the cause to be. One crucial factor in decisions both in the delivery room and in the NICU is parental wishes and values. If *parents* find it more difficult to agree to stopping of treatment in intensive care, that would support a different (more stringent) approach to EOL decisions in the NICU. However, professional reluctance to withdraw treatment may be a form of cognitive bias (labelled “withdrawal aversion”) [40]. If withdrawing and withholding treatment are actually ethically equivalent, it may be important that professionals overcome such aversion and offer discontinuation of treatment in situations where withholding would be permissible. Two practical heuristics are described in Box 1. The second of these, applied to Case 3, suggests that if doctors would have been prepared to withhold active resuscitation in the delivery room for the infant (had antenatal counselling been possible), they should now be prepared to offer withdrawal of treatment to the infant.

Box 1 **Overcoming withdrawal aversion for extremely premature infants**

**Table:** 

1. The equivalence test: If a preterm infant (P1) is currently receiving life-sustaining treatment (e.g. on a ventilator) and there is a question about withdrawing treatment—imagine that another infant (P2) were to present tomorrow with identical features to P1 (e.g. illness, prognosis, parental wishes, etc.) but is not yet ventilated. Would you be prepared to withhold treatment from P2? If so, on the basis of ethical consistency you should be prepared to withdraw treatment today from P1.
2. The “if I’d only known” test. If a preterm infant, P is currently receiving treatment, think back to decisions at the time of birth (had it been possible to provide antenatal counselling). Imagine that you knew then what you know now about the patient (in terms of response to treatment, complications prognosis, etc.), would you have been prepared to withhold treatment at birth then? If so, you should be prepared to withdraw treatment from P now.

#### 3.1.2 Decisions for EPI versus older infants and children

Whether considering withholding of treatment, or treatment withdrawal, one controversial question is whether the thresholds for treatment decisions for extremely premature infants should be different from those that apply to other patients [41].

Case 4 An appropriate for gestational age 23 week female infant who has received antenatal steroids is estimated, based on relevant national statistics, to have approximately a 50% chance of survival if provided with intensive care in the delivery room, while having a 50% chance of no or only mild impairment if they survive. Most neonatologists would be willing to withhold treatment at parental request in this setting. Would it be regarded as ethical not to embark on life sustaining therapy for an older child with similar prognosis?

There is some evidence that end of life decision-making appears to be different in the paediatric intensive care unit (PICU) compared to the NICU. In a children's hospital in the Netherlands, 25% of deaths in the PICU occurred despite full intensive care, while this applied to only 4% of non-preterm deaths in the NICU (EPI were excluded from the analysis in this study) [42]. The reasons underpinning EOL decisions also appeared to differ: 71% of decisions in the NICU were on the basis of predicted poor quality of life, compared with only 22% in the PICU [42]. Similar findings were observed in a Canadian hospital (study population included EPI): withdrawal of treatment on the basis of quality of life concerns was much more common in the NICU compared to the PICU [43].

At the same time, there is evidence that attitudes to treatment decisions are different for EPI compared to older infants and children. In a series of surveys, involving patients of different ages, Annie Janvier and colleagues found that health professionals in several high income countries reached different judgements about best interests and decisions for EPI [44,45]. For example, in one study, approximately 65% of US health professionals judged it acceptable to withhold treatment in a case like case 4, but only 15% were prepared not to resuscitate a 2 month old infant with bacterial meningitis and identical prognosis [44]. There are different ways of interpreting these findings [45]. One possibility (suggested by Janvier), is that this is evidence of bias against EPI – perhaps as a consequence of attitudes towards the permissibility of abortion, or historic social, cultural or anthropological attitudes towards fetal loss? [46] It may reflect that neonatologists potentially feel more guilty or responsible if their patients survive with severe disabilities [45]. However, there are other possibilities. For example, it could be that the neonatologists are correct to permit parental discretion in the face of such uncertainty, and paediatricians caring for older children should be more willing to contemplate end of life decisions in the face of high mortality and uncertain outcome [6]. Another possibility is that there are relevant contextual differences. The burden of treatment for extremely preterm infants and their families is considerable – involving many weeks and sometimes months of intensive care, frequent invasive and painful procedures. The duration of intensive care stay is usually significantly shorter for older children with critical illness. Where term or older infants do have an anticipated prolonged intensive care stay with high burden of treatment and uncertain outcome (e.g. hypoplastic left heart syndrome, or tracheostomy and long-term ventilation), in our experience it is often judged to be reasonable to withhold therapy and provide EOLC should parents wish this.

### 3.2 End of life care

Where end of life care is provided for EPI, it is common for infants to be on the ventilator and to have mechanical ventilation withdrawn. That leads to some differences in EOLC in the NICU compared to the described practice in the delivery room. First, such infants usually have vascular access through which medication could be given. Second, infants will often already be receiving analgesic or sedative medication at the time of reaching an end of life decision. Third, the nature of the conditions that infants are experiencing might well be anticipated to cause significant discomfort or distress (e.g. respiratory failure or necrotising enterocolitis).

Overall, the nature of EOLC in the NICU is likely to be similar for EPI as for other infants. Non-pharmacological interventions should always be employed, for example cuddles, ensuring a calm environment with minimal noise and light stimuli, non-nutritive sucking with a dummy, music, and positioning. Available evidence suggests that opioid use is common in EPI [20,47]. One study found that the attitudes of NICU and PICU nurses were similar towards end of life opioid use, as were rates of use in dying patients in the two locations [48]. As noted earlier, buccal and intranasal preparations are possibilities for rapid symptom management, and may be used in addition to longer acting medication via the enteral route or subcutaneous infusion. Specialist palliative care teams may be consulted to advise on symptom management plans in this context.

One controversial area of end of life care is the active ending of life. Does this occur for EPI? One of the few jurisdictions to explicitly permit active ending of life in newborn infants is the Netherlands, where the so-called Groningen protocol has set out conditions in which physicians would not be prosecuted [49]. However, reports suggest that this is performed extremely rarely in infants, and not at all in EPI. One reason may be that this protocol was designed for infants not dependent on life-support, while virtually all EPI for whom EOL decisions are made will be receiving one or more forms of life-sustaining treatment. However, other practices that overlap with active ending of life may occur for EPI. A recent national study in Belgium indicated that about 8–10% of deaths in infants <26 weeks at birth were accompanied by the use of medication with explicit intention to hasten death (usually analgesic doses) [50]. In another report from the same study, continuous deep sedation was provided in approximately one quarter of deaths of extremely preterm infants [51]. This was lower than the rate in more mature infants.

One area where end of life care may differ for EPI is in the option of transfer to home or hospice for end of life care [52]. This can be an important option for end of life care for newborns as for older patients, though occurs in only a very small proportion of neonatal deaths. In our experience, it is extremely rare for this to be considered in relation to end of life care for EPI. That may reflect the physiological instability of most EPI who are transitioning to end of life care and the short time frame for decisions. In such circumstances, the risk of deterioration during transfer may be significant and transfer may be impractical [52]. However, it is also possible that it is not considered or offered because of lack of familiarity. It would be important to know how often parents would value this option and whether it could logistically be supported more than it is currently. Some parents will value the opportunity to spend time with their baby after death and nursing staff may benefit from training and support in order to facilitate this, and to consider options for location of after death care.

## 4 Conclusions

### 4.1 Suggestions for practice and research

It is difficult to make confident recommendations about provision of EOLC in the delivery room given the paucity of available data. It would be helpful to have studies that prospectively assess such infants (for example using pain scores), and record the perceptions of both attending health professionals and parents about the infant's symptoms. Furthermore, it would be extremely helpful to have studies of the impact of different approaches to symptom management.

In the meantime, one practical approach would be to consider using either buccal diamorphine, oral morphine or intranasal fentanyl where EOLC is being provided in the delivery room, non-pharmacological measures have not been successful and either health professionals or parents are concerned that the infant is suffering. Since midwives may not have experience of providing palliative care and administering opioids to dying newborns, this indicates a potential role for neonatal teams.

For infants in the NICU, there is a need to improve prognostication, but also to support end of life decision-making, addressing the concerns of health professionals and parents. It would be helpful to have prospective studies evaluating different approaches to symptom management in EPI receiving EOLC. There is also a need to explicitly consider the question of place of death and after death care, and (in at least some cases) offer parents the option of transfer to home or hospice.

## Funding

This research was funded in whole, or in part, by the 10.13039/100010269Wellcome Trust [203132/Z/16/Z]. The funders had no role in the preparation of this manuscript or the decision to submit for publication. For the purpose of open access, the author has applied a CC BY public copyright licence to any Author Accepted Manuscript version arising from this submission.

## Declaration of competing interest

The authors have no conflicts of interest.
